# A Randomised Controlled Trial of Triple Antiplatelet Therapy (Aspirin, Clopidogrel and Dipyridamole) in the Secondary Prevention of Stroke: Safety, Tolerability and Feasibility

**DOI:** 10.1371/journal.pone.0002852

**Published:** 2008-08-06

**Authors:** Nikola Sprigg, Laura J. Gray, Tim England, Mark R. Willmot, Lian Zhao, Gillian M. Sare, Philip M. W. Bath

**Affiliations:** Stroke Trials Unit, Institute of Neuroscience, University of Nottingham, Nottinghamshire, United Kingdom; Duke Clinical Research Institute, United States of America

## Abstract

**Background:**

Aspirin, dipyridamole and clopidogrel are effective in secondary vascular prevention. Combination therapy with three antiplatelet agents might maximise the benefit of antiplatelet treatment in the secondary prevention of ischaemic stroke.

**Methodology/Principal Findings:**

A randomised, parallel group, observer-blinded phase II trial compared the combination of aspirin, clopidogrel and dipyridamole with aspirin alone. Adult patients with ischaemic stroke or transient ischaemic attack (TIA) within 5 years were included. The primary outcome was tolerability to treatment assessed as the number of patients completing randomised treatment. Recruitment was halted prematurely after publication of the ESPRIT trial (which confirmed that combined aspirin and dipyridamole is more effective than aspirin alone). 17 patients were enrolled: male 12 (71%), mean age 62 (SD 13) years, lacunar stroke syndrome 12 (71%), median stroke/TIA onset to randomisation 8 months. Treatment was discontinued in 4 of 9 (44%) patients receiving triple therapy vs. none of 8 taking aspirin (p = 0.08). One recurrent stroke occurred in a patient in the triple group who was noncompliant of all antiplatelet medications. The number of patients with adverse events and bleeding complications, and their severity, were significantly greater in the triple therapy group (p<0.01).

**Conclusions/Significance:**

Long term triple antiplatelet therapy was asociated with a significant increase in adverse events and bleeding rates, and their severity, and a trend to increased discontinuations. However, the patients had a low risk of recurrence and future trials should focus on short term therapy in high risk patients characterised by a very recent event or failure of dual antiplatelet therapy.

**Trial Registration:**

Controlled-Trials.com ISRCTN83673558

## Introduction

Increased platelet activity plays a critical role in the pathophysiology of non cardioembolic ischaemic stroke, both in its initial cause and the subsequent risk of recurrence. Several antiplatelet agents have been used to prevent recurrence, [1] these having different mechanisms for inhibiting platelets. The archetypal agent, aspirin (A), irreversibly inhibits cyclo-oxygenase and reduced the relative risk of stroke recurrence by 15–20% across a wide range of trials.[2] Clopidogrel (C, a pro-drug which antagonises ADP receptors), showed a slight benefit over aspirin in a mixed population of patients with vascular disease;[3] no difference was seen in the sub-group of patients with prior ischaemic stroke.[3] Dipyridamole (D), an inhibitor of phosphodiesterase and adenosine uptake by platelets, had comparable efficacy to aspirin in one trial.[4] Dual therapy with the combination of aspirin and dipyridamole was superior to aspirin alone in preventing stroke recurrence [4], [5] and had twice the efficacy of mono-therapy when compared with placebo.[4], [6] Although the combination of aspirin and clopidogrel was superior to aspirin alone in preventing vascular events in cardiac patients with unstable angina or requiring percutaneous coronary intervention, [7], [8] it showed no overall benefit in patients with stable vascular disease or at risk of developing a first vascular event.[9] However, two small trials involving patients at high risk of stroke recurrence suggested that combined aspirin and clopidogrel were superior to aspirin alone.[10], [11]

If two antiplatelet agents are superior to one then three agents with differing modes of action might be better still, providing the risk of bleeding does not become excessive. In laboratory studies, we found that the combination of aspirin, dipyridamole and AR-C69931 (a direct-acting antagonist of the ADP receptor) was superior to dual or mono-therapy in inhibiting platelet aggregation and activation, leukocyte activation, and the formation of platelet leucocyte conjugates *in vitro*.[12] However, in normal subjects and patients with prior ischaemic stroke, short-term triple therapy was no more effective than combined aspirin and clopidogrel in moderating leucocyte and platelet function.[13] In addition to antiplatelet effects, these agents have non-platelet effects on thrombosis, inflammation and endothelial function which may also play a part in stroke prevention; for example, dipyridamole reduces circulating von Willebrand factor levels and blood pressure [14], [15] whilst clopidogrel may release prostacyclin and tissue plasminogen activator.[16]

We have previously described the use of open-label triple antiplatelet therapy in patients who have suffered stroke recurrence while taking dual therapy.[17] Here, we report a phase II trial which assessed the safety, tolerability and feasibility of giving triple antiplatelet therapy in comparison with aspirin (the UK standard of care at the time the trial commenced) alone in patients with prior ischaemic cerebrovascular disease.

## Methods

The protocol for this trial and supporting CONSORT checklist are available as supporting information; see Checklist S1 and Protocol S1.

### Participants

Adult patients with ischaemic stroke or transient ischaemic attack (TIA) within the past 5 years were identified and enrolled from Nottingham City Hospital (NCH). Principal exclusion criteria included a history of peptic ulcer disease; prior cerebral haemorrhage; anaemia; thrombocytopaenia; hypersensitivity or intolerance to any of the antiplatelet agents; need for dual antiplatelet therapy, anticoagulation or non-steroidal anti-inflammatory drugs; or involvement in another clinical trial. Full written informed consent was obtained from all patients prior to randomisation. Patients who had not previously received dipyridamole were given open label modified release dipyridamole 200 mg (with aspirin) for a period of 2 weeks; only those patients without significant adverse events continued to randomisation which was performed following a three week washout period when only aspirin was taken.

### Ethics

The study was approved by the Nottingham Local Research Ethics Committee (October 2001), had a ‘Medicines and Healthcare products Regulatory Agency’ Clinical Trial Authorisation (August 2001) and International Trial Number (ISRCTN83673558), and was performed according to the Declaration of Helsinki and the International Conference on Harmonisation of Good Clinical Practice guidelines. All patients gave written informed consent; if the patient was not competent to give assent (e.g. dysphasia, confusion) a relative was approached to give assent.

### Interventions

Patients were randomised to receive either open-label combined aspirin (A, 75 mg od), clopidogrel (C, 75 mg od), and modified release dipyridamole (D, 200 mg bd) (ACD) or aspirin (75 mg od) alone (no dipyridamole or clopidogrel placebo) on a 1∶1 ratio. Mono-therapy with aspirin was chosen as the active comparator since this was the standard of care at the time of trial design; the comparison of triple with mono therapy meant that a smaller sample size was possible since any difference in the primary outcome (tolerability) was likely to be maximised.

### Objectives

The trial comprised a prospective, randomised, single-centre, observer-blinded, aspirin-controlled phase II trial of triple antiplatelet therapy in patients with previous ischaemic stroke or transient ischaemic attack. The aim was to test the safety, tolerability and feasibility of combined aspirin, clopidogrel and dipyridamole; additional haematological and haemodynamic measures were also made.

### Outcomes

The primary outcome was tolerability of treatment assessed as the number of patients completing randomised treatment at final follow up. Secondary outcomes included safety (mortality), serious adverse events (SAEs), stroke recurrence (ischaemic or haemorrhagic), and extra-cranial bleeding (major and/or minor).

Haematology measures were performed at baseline and 2 weeks after starting treatment using our previously published methodology.[13] Venepuncture was performed after 10 minutes of rest and blood collected into hirudin. Platelet aggregation was performed in whole blood using aliquots stirred with ADP (final concentration 3 µmol/L), collagen (2 µg/mL) or platelet activating factor (PAF, 1 µmol/L). Aggregation was assessed at 4 minutes by counting the number of fixed single platelets relative to the starting platelet count using an Ultra-Flo 100 Whole Blood Platelet Counter.[13]

Platelet-leucocyte conjugate formation and leucocyte CD11b expression (a measure of leucocyte activation) in response to ADP, collagen or PAF were determined by flow cytometry. 100 µl aliquots of the blood were treated with Erythrolyse solution for 10 min at room temperature, centrifuged and then washed.[13] 30 µl aliquots were incubated with saturating concentrations of anti-CD14:PE to identify monocytes and anti-CD42a:FITC to identify platelets bound to monocytes and/or neutrophils. Platelet-monocyte and platelet-neutrophil conjugates were then quantified by flow cytometry. Further 30 µl aliquots were incubated with anti-CD14:PE and anti-CD11b:FITC to detect leucocyte activation.[13]

Platelet-leucocyte conjugates and leucocyte CD11b expression were quantified using a FACScan flow cytometer (Becton Dickinson) equipped with a 5 W laser operating at 15 mW power and a wavelength of 488 nm, and connected to an Apple Mac computer.[13] Leucocytes were monitored using forward and side light scatter, and fluorescence. Monocytes were differentiated from other leucocytes by their CD14:PE positivity. Platelet-leucocyte conjugates are reported as the median CD42a fluorescence of the leucocyte populations. CD11b expression is reported as the median CD11b:FITC fluorescence of the leucocyte populations.[13]

Full blood count measures for haemoglobin, and red cell, platelet and white cell count were performed at baseline and final follow-up by Nottingham City Hospital's clinical haematology laboratory.

Since dipyridamole has vasoactive properties, peripheral blood pressure (systolic BP, diastolic BP, postural drop) and heart rate were measured with a validated digital readout oscillometric device (Omron HEM-705CP, Illinois, US) at baseline, 2 weeks, 3 months and final follow up, with measurements performed blinded to treatment assignment.

### Sample size

A sample size of 51 was required assuming the number of subjects completing treatment to the end of the trial was 55% in the ACD group and 90% with aspirin alone, with significance 0.05, power (1-beta) 0.80, and 1∶1 randomisation (ACD:A). The assumptions were based on previous experience with triple antiplatlet therapy and, in particular, the propensity for research subjects to develop headache with dipyridamole. No interim analysis was planned.

### Randomization—Sequence generation

Randomisation was performed following consent using computer minimisation on age (>70 years), delay from ischaemic event, ischaemic event (stroke or TIA) and baseline systolic blood pressure (SBP). This approach ensures concealment of allocation and adds to statistical power.[18]

### Randomization—Allocation concealment

Assignment was changed randomly for 10% of patients to prevent researchers from trying to guess the next allocation.

### Randomization—Implementation

Patients were identified and enrolled by research nurses (with consent obtained by clinical research fellows); treatment assignment was performed by a third party.

### Blinding

The interventions were unblinded to both patients and treating staff. However, patient assessments on and at the end of treatment, adjudication of events, and assessment of haematology and haemodynamic measures, were performed blinded to treatment assignment by trained staff.

### Statistical methods

Data are presented as mean (standard deviation, SD), median (interquartile range, IQR) or number (%). Data were compared using Fisher's Exact test, repeated measures ANOVA, or ANCOVA with adjustment for baseline values. Adverse events were re-coded as ordinal variables, with data scored as follows: no bleed = 0, minor bleed = 1, major bleed = 2 and no adverse event = 0, adverse event = 1, non fatal serious adverse event = 2, death = 3. These ordinal scores were then analysed by treatment group using a Mann-Whitney U test.[19] Analyses were performed using SAS (version 8) and were by intention-to-treat; statistical significance was taken at p<0.05. No adjustments for multiple comparisons were made.

## Results

### Participant flow and recruitment

**Figure 1:**
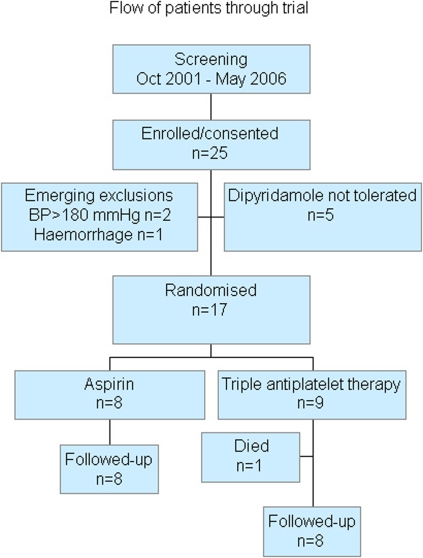
Flow of patients through trial.

Seventeen patients were enrolled between 2002 and 2006 (figure 1). The trial was stopped prematurely following publication of the ESPRIT trial which confirmed ESPS II in showing that combined aspirin and dipyridamole is superior to aspirin alone in preventing recurrent stroke and other vascular events.[4], [5] Additionally, the UK National Institute of Clinical Excellence recommend this combination as first line therapy for patients with prior ischaemic cerebrovascular disease.[20] As such, we considered it unethical to continue randomising patients to receive mono-therapy with aspirin.

### Baseline data and numbers analysed

9 patients were randomised to triple therapy and 8 to aspirin. Both groups were matched for age, baseline event (stroke or TIA) and stroke syndrome (table 1). Patients were enrolled at an average of 12 months (range 1.5–30) after their stroke or TIA. The total exposure time to treatment was 282 months (triple 138, aspirin 144 months).

**Table 1:** Baseline characteristics of patients. Number (%), mean (standard deviation), or median [interquartile range].

	Aspirin	Triple
Number	8	9
Sex, male (%)	6 (75)	6 (67)
Age (years)	61 (14)	63 (12)
Time to randomisation (months)	10 [18]	8 [5]
Randomisation event (%)		
Stroke	7 (88)	8 (89)
Transient ischaemic attack	1 (13)	1 (11)
Lacunar syndrome (%)	5 (63)	7 (78)
Location, left hemisphere (%)	4 (50)	3 (33)
Previous family history of stroke (%)	3 (38)	4 (44)
Previous stroke or TIA (%)	1 (13)	1 (11)
Hypertension (%)	5 (63)	6 (67)
Diabetes (%)	1 (13)	1 (11)
Hyperlipidaemia (%)	6 (75)	4 (44)
Atrial fibrillation (%)	0 (0)	0 (0)
Ischaemic heart disease (%)	1 (13)	0 (0)
Peripheral vascular disease (%)	1 (13)	1 (11)
Current smoker (%)	3 (38)	6 (67)
Systolic blood pressure (mmHg)	132 (20)	148 (21)
Diastolic blood pressure (mmHg)	78 (12)	83 (9)
Postural blood pressure drop (mmHg)	6 (18)	−7 (14)
Heart rate (bpm)	70 (8)	76 (11)

### Outcomes and estimation

Treatment discontinuations numbered 4 (44%) in the triple therapy group: bruising 1 patient, gastrointestinal bleeding 2, and non-compliance to all trial drugs 1; no patients stopped mono-therapy (aspirin alone) early (p = 0.08) (table 2)

**Table 2:** Adverse Events by treatment assignment and patient.

Treatment	Trial no.		Timing (months)	Serious Adverse Event	Relationship of event to treatment
Triple	1	Diuretic-induced hyponatraemia/collapse	9	SAE	Not related
		Gastritis (no gastroscopy) with anaemia†	9		Probable
	4	Shortness of breath	0.5		Unlikely
	6	Bruising	3		Probable
	10	Upper gastrointestinal bleed†	6	SAE	Probable
		Acute myeloid leukaemia (when off treatment)	15	SAE (fatal)	Not related
	11	Prolonged bleeding at a skin cut	0.5		Probable
		Retinal vein occlusion	15		Unlikely
	12	Recurrent minor stroke of unknown type (when non-compliant with all medications†)	12		Possible
	14	Bruising	3		Probable
	17	Bruising following minor trauma	0.5		Possible
		Headache	3		Probable
		Bruising†	3		Probable
Aspirin	13	Tired	0.5		Unlikely
	15	Diarrhoea, stomach cramps	2		Possible

† Leading to treatment cessation.

SAE: serious adverse event.

### Ancillary analyses

In comparison with aspirin, triple therapy appeared to exhibit enhanced antiplatelet effects manifest as reduced collagen-induced platelet aggregation and monocyte activation, and ADP-induced platelet-monocyte conjugation (table 3). Haemoglobin levels at final follow up did not differ between the triple therapy and aspirin groups (13.4 vs. 13.9 g/dL, p = 0.76).

**Table 3:** Haematological measures at week 2; mean (standard deviation); comparison by ANCOVA with adjustment for baseline values.

	Agonist	Aspirin	Triple	ANCOVA p value
Aggregation (at 4 mins)	ADP	42.6 (17.9)	34.1 (24.4)	0.38
	Collagen	84.0 (5.5)	69.9 (11.1)	0.05
	PAF	26.2 (13.4)	42.1 (21.9)	0.89
Monocyte activation (CD11b)	ADP	89.2 (10.8)	80.4 (12.6)	0.17
	Collagen	165.0 (107.8)	95.7 (45.5)	0.02
	PAF	159.8 (110.6)	144.0 (57.7)	0.20
Platelet-monocyte conjugates	ADP	117.8 (76.6)	74.1 (34.4)	0.04
	Collagen	97.4 (3.8)	97.1 (1.9)	0.23
	PAF	90.8 (6.4)	93.0 (7.0)	0.23

No significant difference in systolic and diastolic blood pressure, postural drop in blood pressure, or heart rate were present between triple therapy and aspirin. There was a non-significant trend to a reduced rate pressure product over time in the triple therapy group (table 4).

**Table 4:** Haemodynamic measures; mean (standard deviation); comparison by repeated measures ANOVA (baseline, 2 weeks, 3 months, final follow up).

	2 weeks		3 months		Final follow up		P value
	Aspirin (7)	Triple (9)	Aspirin (8)	Triple (9)	Aspirin (8)	Triple (8)	
Systolic BP (mmHg)	129 (21)	146 (17)	131 (15)	140 (16)	147 (21)	139 (16)	0.42
Diastolic BP (mmHg)	73 (10)	77 (13)	80 (13)	77 (13)	79 (10)	79 (9)	0.79
Pulse pressure (mmHg)	56 (15)	68 (17)	52 (13)	63 (16)	68 (12)	61 (18)	0.39
Postural drop systolic BP (mmHg)†	2 (15)	−12 (7)	−4 (18)	−11 (11)	−13 (15)	−1 (24)	0.37
Heart rate (bpm)	71 (12)	82 (15)	72 (11)	74 (12)	76 (10)	72 (13)	0.55
Rate-pressure product (mmHg.bpm)	9105 (1959)	11948 (2565)	9399 (1393)	10421 (1962)	11085 (1823)	9919 (1915)	0.19

† Measured as standing systolic-sitting systolic.

### Adverse events

**Figure 2:**
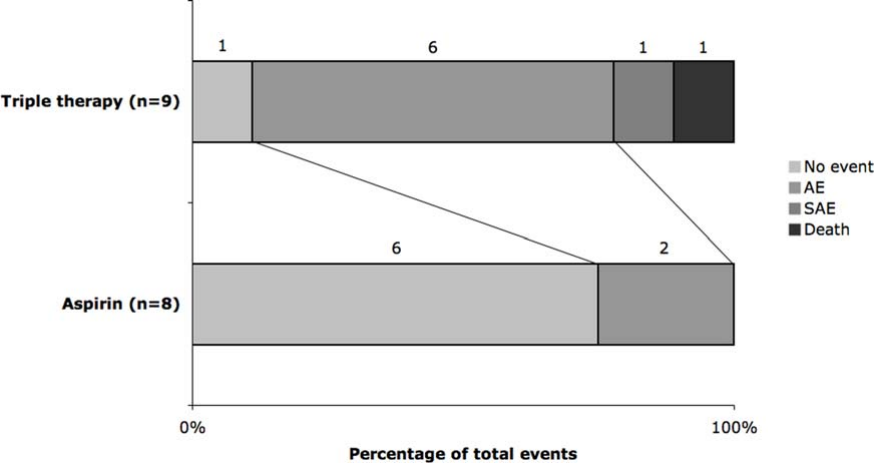
Frequencies of adverse events in aspirin and triple therapy groups.

One patient died in the triple therapy group of acute myeloid leukaemia; no patients died in the aspirin group. When bleeding events were analysed as ordinal data (no bleed, minor bleed, major bleed)[19], significantly increased rates were seen in the triple therapy group (p<0.01). Similarly, there was a significant increase in the number and severity of adverse events (ordered as no event, adverse event, non-fatal serious adverse event, death) in the triple group (p<0.01) (table 2 and figure 2). Only one of the SAEs was thought to be related to treatment. There was a non-significant difference in efficacy between treatment groups (p = 0.53); one recurrent stroke (non-disabling) occurred in a patient randomised to triple therapy who was noncompliant of all three antiplatelet agents.

## Discussion

### Interpretation

This phase II trial has found that treatment with triple antiplatelet therapy was associated with a trend to increased treatment drop-outs as compared with aspirin alone (treatment discontinuation 44% in the triple group versus 0% in the aspirin group) in patients at low risk of stroke recurrence. 3 of the 4 patients who ceased therapy prematurely did so with symptoms related to bleeding or bruising; 1 of these events was defined as serious. However, long term aspirin monotherapy may be safer with less (and less severe) bleeding and fewer (and less severe) adverse events than long term triple antiplatelet therapy. The finding of an increase in bleeding with triple therapy is supported by laboratory testing *ex vivo* where reduced platelet aggregation (as well as reduced monocyte activation and the formation of platelet-monocyte conjugates) was seen with triple therapy as compared with aspirin. Our previous work *in vitro* suggests that the measured antiplatelet effects will largely have been due to the combination of aspirin and clopidogrel rather than dipyridamole.[13] Furthermore, increased bleeding has been seen in trials of combined aspirin and clopidogrel [7]–[9] but not combined aspirin and dipyridamole.[4], [5]

Dipyridamole has vasoactive properties due to its effect as an inhibitor of phosphodiesterase and red cell adenosine uptake. As such, it lowers blood pressure, particularly when given intravenously [21] but only modestly when given orally.[14] Although no hypotensive effects were seen in this study, a trend to a reduced rate pressure product was apparent in patients randomised to triple therapy as compared with aspirin alone. Since aspirin and clopidogrel do not have vasoactive properties, it is most like that dipyridamole contributed to any vasoactive effects seen here.

The major limitation of this study is its unintended small size resulting from having to stop recruitment early. Recruitment was halted prematurely with only 17 of the planned 51 patients recruited following the publication of ESPRIT;[5] together, ESPS II and ESPRIT show that the combination of aspirin and dipyridamole is superior to aspirin alone in preventing recurrent stroke and other vascular events [4], [5] thereby making it unethical to continue randomising patients to aspirin mono-therapy. As a result, many of the analyses are very underpowered such as the neutral findings for effects of dipyridamole on haemodynamic measures, these probably reflecting a type II error. A further important criticism is that the recruited patients were at low risk of recurrence; specifically, they were younger than most patients presenting with stroke (mean age 62 years) and were enrolled up to 5 years after the index event (median 12 months) whereas the risk of recurrence is highest in the first few hours and days after ictus. Furthermore, the majority of patients presented with a lacunar stroke, again a group at relatively low risk of recurrence. Hence, the balance between risk of recurrence (low) and bleeding (relatively high on triple therapy) was probably inappropriate, a problem also seen in MATCH and CHARISMA.[9], [22] Future trials of triple antiplatelet therapy should focus on high risk patients with recent events or who have experienced a further event on dual antiplatelet treatment (so-called ‘failure’); such studies might limit the length of treatment to cover the period of maximum risk of recurrence, e.g. for the first three months after ictus.

### Generalizability

Analogous trials of triple antiplatelet therapy have been done in other groups of patients at high risk of vascular events. These were analysed in a recent systematic review comparing 23 completed randomised controlled trials assessing various classes of antiplatelet therapy; in comparison with aspirin-based dual or mono therapy, triple therapy significantly reduced vascular events, myocardial infarction and death (in high risk vascular syndromes) offset with an increased bleeding risk. [Geeganage, Wilcox, Bath, unpublished data] There was, however, insufficient data on ischaemic stroke highlighting the need for further research in this area.

### Overall evidence

Despite the above limitations, this study is the first long-term randomised controlled trial of combined aspirin, clopidogrel and dipyridamole after stroke and provides valuable safety and feasibility data, and guidance on how future trials should be performed. Further evidence is now required regarding the safety and efficacy of triple therapy for the secondary prevention of stroke in patients at high risk of recurrence. This approach may be useful in those patients who have a recurrent stroke on dual therapy.[17] In light of ESPS 2 and ESPRIT [4], [5] confirming the superiority of aspirin and dipyridamole versus aspirin alone, future trials will need to compare the effect of adding clopidogrel to dual therapy versus dual therapy alone.

## Supporting Information

Checklist S1 Consort Checklist.
(0.07 MB DOC)

Protocol S1 Trial Protocol.
(0.03 MB DOC)
