# Supplementary material for: A Randomised Controlled Trial of Triple Antiplatelet Therapy (Aspirin, Clopidogrel and Dipyridamole) in the Secondary Prevention of Stroke: Safety, Tolerability and Feasibility
Source: PLoS One. 2008 Aug 6;3(8):e2852. doi: 10.1371/journal.pone.0002852 (PMC2481397; doi:10.1371/journal.pone.0002852)
Supplement: Protocol S1 — Trial Protocol. (0.03 MB DOC) [file pone.0002852.s002.doc]

COMBINED ASPIRIN, CLOPIDROGREL AND DIPYRIDAMOLE VERSUS ASPIRIN ALONE IN STROKE SECONDARY PREVENTION; A SAFETY TOLERABILITY AND FEASIBILTY STUDY

BACKGROUND

Stroke is a leading cause of long term disability and death around the world. Increased platelet activity plays a critical role in the physiology of stroke. The key medical strategy for the secondary prevention of stroke is anti-platelet therapy. Several antiplatelet agents have been used which inhibit different pathways of platelet activation. The earliest agent aspirin, a cyclo-oxygenase inhibitor, showed a reduction of relative risk for stroke recurrence of approximately 25% compared with placebo1. The newly developed ADP receptor blocker clopidrogrel, showed a slight benefit over aspirin2. Dipyridamole, a phosphodiesterase inhibitor and modulator of adenosine uptake, is an effective preventative agent when used alone after stroke3. Furthermore the combination of aspirin and dipyridamole was superior to either agent alone3. The European Atrial Fibrillation Trial4 found that warfarin was superior to aspirin in the secondary prevention of stroke in patients with cardioembolic cerebral ischaemia due to atrial fibrillation. However, clinical practice reveals that between 50-66% of stroke survivors are unable to take warfarin, mostly for reasons of contraindication or problems with regular monitoring of anticoagulation. We hypothesise that combination therapy with three antiplatelet agents that act through different mechanisms may maximise the benefit of antiplatelet treatment in the secondary prevention of stroke, both in patients with sinus rhythm and those with stroke who cannot be anticoagulated. In a laboratory study we found that triple antiplatelet therapy was superior to double or monotherapy in respect of inhibiting invitro platelet function, leukocyte function and the formation of platelet leukocyte conjugates5 . Provisional data from an ongoing pilot study in normal subjects and patients with previous ischaemic stroke (ethics no. EC00/08) support the concept that triple therapy is most effective in inhibiting platelet and leukocyte function ex vivo (unpublished data).

AIM

To assess the safety, tolerability and feasibility of administering triple antiplatelet therapy (aspirin, dipyridamole, and clopidrogrel) in comparison with aspirin. This study is the prelude to a large phase 3 trial to be submitted to the MRC for funding.

DESIGN

Prospective, pilot, randomised, parallel group trial.

INTERVENTIONS

Combined aspirin (75mg od, A) dipyridamole MR (200mg bd, B) and clopidrogrel (75mg od, C) versus aspirin (75mg once daily, A) alone.

ELIGIBILTY

Inclusion criteria for patients

Age 18+

Ischaemic stroke on CT/MRI within 5 years

Previous TIA within 5 years

Written informed consent from patient or assent from a legally acceptable representative

In sinus rhythm or in atrial fibrillation but not suitable for anticoagulation

Exclusion criteria

Thrombocytopenia (platelet count<100) and or anaemia (Hb<10)

Severe hypertension SBP >180 or DBP>110 mmHg

Previous cerebral haemorrhage

Hypersensitivity or intolerance to aspirin, dipyridamole or clopidrogrel

Any history of peptic ulcer or other gastrointestinal bleeding

Severe concomitant medical conditions including AIDS or cancer

Pregnancy or breast feeding

Patients needing or already receiving anticoagulant or NSAIDS other than aspirin therapy

Involvement in another secondary prevention drug trial

Not available for follow up eg. overseas visitor

METHOD

51 patients with a previous diagnosis of TIA or ischaemic stroke and in sinus rhythm within 5 years will be recruited into the trial. All those patients who have not previously received and tolerated dipyridamole will be given open label dipyridamole 200mg MR for a period of 2 weeks before randomisation (as per ref. EC00/08). This will be in addition to any aspirin therapy that these patients are already on. Only those patients that have completed 2 weeks without significant adverse events will continue into the trial. There will then be a 3 week washout period before participants are randomised to receive either aspirin 75mg daily or triple antiplatelet therapy. Patients with AF who are not being anticoagulated will also be enrolled in a similar manner. It is expected that the maximum follow up period for the first patient will be 15 months (maximum 2 years), ie. recruitment will take place over 12 months. Triple therapy will consist of a combination of aspirin (75mg daily), dipyridamole MR (200mg twice daily) and clopidrogrel (75mg daily). Prior to starting, all patients will have data collected relating to general demographics, risk factors, medications, smoking history, postural blood pressure measurement. In addition, a medical examination will be undertaken to find any clinical contraindications and baseline venous blood samples will be collected for the lab measures detailed below. Patients will then commence the randomised treatment regimen and be followed up after 2 weeks and then every three months (after the first 3 month follow up patients will be followed up by telephone every third month, the final follow up will be by a clinic visit). Blood tests will be repeated at 2 weeks and three months. Postural BP and pulse rate will be measured at 2 weeks, 3 months and final follow up. Screening for adverse events will occur at every follow up point. The study will continue until the last recruited patient has had their first 3 months follow up.

OUTCOME MEASURES

Primary

Number of subjects completing randomised treatment to final follow up.

Secondary

Recurrent ischaemic stroke

Recurrent TIA

Intracerebral haemorrhage

Major extracranial bleeding (clinically overt bleeding associated with one or more of; Transfusion of at least 2 units of red cells, a fall in Hb of at least 2g/l or bleeding leading to cessation of treatment.)

Minor extracranial bleeding (epistaxis, purpura)

Sitting and standing blood pressure, heart rate at 2 weeks, 3 months and follow up.

Presence of headache

Lab Measures

Platelet Activity and leukocyte function (as in protocol approval EC00/08)

-Full blood count (Baseline, 3, 12 and 24 months)

-Platelet count

-Platelet aggregation in whole blood by single platelet counting. (hirudin as anticoagulation, ADP, PAF , collagen and AA as agonists).

-Mean platelet volume (in whole blood)

-Activated marker on platelet. (P-selectin CD62p by flow cytometry from whole blood).

-White cell platelet aggregates (CD14 on monocytes, CD11b on neutrophils and monocytes, CD42a on platelets by flow cytometry.)

SAMPLE SIZE

Assuming an overall significance (alpha) of 0.05, power (1-beta) = 0.80, 2:1 randomisation (ACD:A), and number of subjects completing treatment to end of trial is 60% in ACD group and 89% in A group3 , the required size would be 51 patients, with 17 in the aspirin group and 34 in the ACD group. Additional patients with atrial fibrillation will be enrolled during the duration of the trial up to a maximum of 30 (ACD:20, A10).

RANDOMISATION

By stratification (sinus rhythm versus atrial fibrillation) and minimisation (age, gender, time from stroke, systolic blood pressure)

REFERENCES

1 Collaborative overview of randomised trials of antiplatelet therapy, prevention of death, myocardial infarction and stroke by prolonged anti platelet therapy in various categories of patients. BMJ 1994; 308: 81-106

2 CAPRIE Steering Committee: A Randomised, blinded, trial of clopidrogrel verses aspirin in patients at risk of ischaemic events (CAPRIE). Lancet 1996; 348:1329-1339.

3 Diener HC, Cunha L Forbes C, Sivenius J Smets P, Lowenthal A: European Stroke Prevention Study 2. Dipyridamole and acetylsalicylic acid in the secondary prevention of stroke. J Neurol Sci 1996; 143: 1-13

4 EAFT (European Atrial Fibrillation Trial) Study Group. Secondary prevention in non-rheumatic atrial fibrillation after transient ischaemic attack or minor stroke. Lancet 1993;342:1255-1262.

5 Zhao L, Bath P, Heptinstall S. Effects of combining three different antiplatelet agents on platelets and leukocytes in whole blood in vitro. Br J Pharmacol; 2001 in press.
